# Home environment and nutritional status mitigate the wealth gap in child development: a longitudinal study in Vietnam

**DOI:** 10.1186/s12889-023-15156-2

**Published:** 2023-02-08

**Authors:** Lan Mai Tran, Phuong Hong Nguyen, Melissa F. Young, Usha Ramakrishnan, Harold Alderman

**Affiliations:** 1 Emory University, Atlanta, GA USA; 2 International Food Policy Research Institute (IFPRI), 1201 I Street, 20001 Washington, NW, DC USA; 3 Thai Nguyen University of Pharmacy and Medicine, Thai Nguyen, Vietnam

**Keywords:** Child development, Early and middle childhood, Home quality environment, Inequity, Mitigating factors, Wealth gaps

## Abstract

**Background:**

Inequity in child development is found at early age, but limited evidence exists on whether these gaps change over time and what are the mediators.

**Objective:**

We aim to (1) quantify wealth related gaps in cognitive and socio-emotional development in early and middle childhood; (2) examine how these gaps were mitigated by maternal, child factors and home environment.

**Methods:**

We assessed the offspring of women who participated in a randomized controlled trial of preconception micronutrient supplementation in Vietnam (n = 1599). Child development was measured by the Bayley Scales of Infant Development-III (at 1-2y) and the Wechsler Intelligence Scale for Children®—IV (at 6-7y). We used multivariable regression to estimate the changes in wealth gaps for child development over time, adjusting for potential factors that potentially influence cognitive development.

**Results:**

We found significant wealth gaps in cognitive development during early childhood (gaps between top and bottom quintiles: 0.5 SD); these gaps increased substantially in middle childhood (0.9 SD). Wealth disparity in social emotion did not change over time (0.26–0.28 SD). Maternal factors, quality of home environment, and child nutritional status mitigated the wealth gap in cognitive development (7-42%) in early childhood. The contribution of these mitigating factors was smaller in middle childhood (2- 15%). Wealth gap in social emotion reduced by 13% and 43% among children with better nutritional status at 2y and higher quality of home environment at 6-7y, respectively.

**Conclusion:**

Interventions focusing on improving quality of home environment, maternal education, wellbeing, and child nutrition status may help reduce developmental deficits associated with poverty.

**Supplementary Information:**

The online version contains supplementary material available at 10.1186/s12889-023-15156-2.

## Introduction

The cumulative impact of health, nutrition, care, and safety risk factors beginning before conception and continuing throughout gestation and early life can set back brain development as well as physical growth. A recent estimate indicates that these challenges may affect up to 43% of children in low- and middle- income countries (LMICs) [1]. These missed opportunities for child development are costly not merely in terms of unrealized potential productivity of the next generation but also in terms of equity [2].

Poor nutrition during the first 1000 days of life can negatively impact cognition by affecting the development of the nervous system and may have long-lasting impacts on learning outcomes [3–8]. Child stunting (height-for-age z-score < 2SD) at two years of age, a marker of nutritional deprivation in many LMICs, has been associated with poorer cognitive, education, and economic outcomes and is often considered as one the best predictors for human capital [9]. Studies have also shown that later catchup growth in children who experienced early growth restriction often does not help attenuate the long-term losses in cognitive outcomes [10]. The effects of poverty and/or inadequate stimulation on brain development seem to persist through the preschool years [11]. In contrast, interventions to improve the quality of childcare can offset some of the negative consequences of early undernutrition [2, 12]. The associations between poverty and reductions in white and cortical gray matter, hippocampal and amygdala volumes were also mediated by high quality early childhood care [11].

Caregiving includes the provision of proper health and nutrition as well as learning opportunities within a safe and secure environment and may manifest in cumulative cognitive and socioemotional outcomes over the life course [1]. Caregiving in turn is influenced by the economic, cultural, political and climatic context of the community as well as the enabling environment. This environment is multifaceted and determined by socioeconomic status, parental education, family environment, and access to timely and high quality health services such as prenatal care [1]. This perspective also helps identify the most effective times for interventions over the life course and provides a basis for understanding the need for comprehensive programs that transition over stages in a child’s development.

Better health [or skills] a child obtains from parental investments in early life may facilitate acquiring even higher levels of health [skills] later [12, 13]. In particular, better health [skills] in one period could lead to greater returns to subsequent parental and social investments including schooling. Thus, moderate deficits during early life – often reflecting social-economic conditions in the household - might lead to greater differences in schooling and other outcomes thereafter. Alternatively, interventions and/or investments that a child receives in a later period might mitigate earlier disparities. For example, children with limited opportunities in their home environments may benefit from preschool programs more than other children. Similarly, nutrition programs like the provision of micronutrients to preschool children may have greater impacts in low resource settings [14]. Thus, it remains an empirical question as to the direction as well as the magnitude of changes in initial poverty related impacts on cognitive development over the preschool period.

Differences in indicators of nutrition and cognitive development across income strata have been well documented; somewhat more noteworthy is the fact that these differences are found at very early ages [2, 15]. Less is known regarding whether these wealth gaps widen or narrow over time and if they do change, what are the influencing factors. While some studies show similar proportional gaps over time for cognitive development between children from the richest and poorest households [15, 16], others find reducing wealth gradients can narrow the cognitive gap [17–20]. The factors that contribute to changes in wealth gaps however are generally country-specific and vary between populations. For example, caregivers’ education has been found to be a main mediating factor in the Young Lives Project in 4 countries (Peru, India, Vietnam, Ethiopia) [16] as well as in a study from Bangladesh [21], but the size of the effect varies widely (from 30 to 80%). Home stimulation was also a significant mitigating variable and explained up to 50% of the wealth effects in community-randomized water and sanitation trials India, Indonesia, Peru, and Senegal [17]. A similar approach was used to study disparities in cognitive development of children associated with wealth in Bogota up to 8 years of age [20]. Again, parental education and home environments were found to be strong mediators while height for age was not. Evidence on wealth gaps related to social-emotional development is limited; we are aware of only two studies in Madagascar and Colombia [18, 22].

The current study builds upon the previous work by indicating the wealth disparities in cognitive and socio-emotional development in the context of Vietnam. Specifically, we aim to: (1) quantify wealth gaps in both cognitive and socio-emotional development in early childhood (at 1y and 2y), and examine whether these gaps persist in middle childhood (at 6-7y); (2) explore how home quality environment, maternal factors, child nutritional status, and preschool attendance would mitigate the wealth gaps.

## Methods

### Data sources and study population

**Fig. 1:**
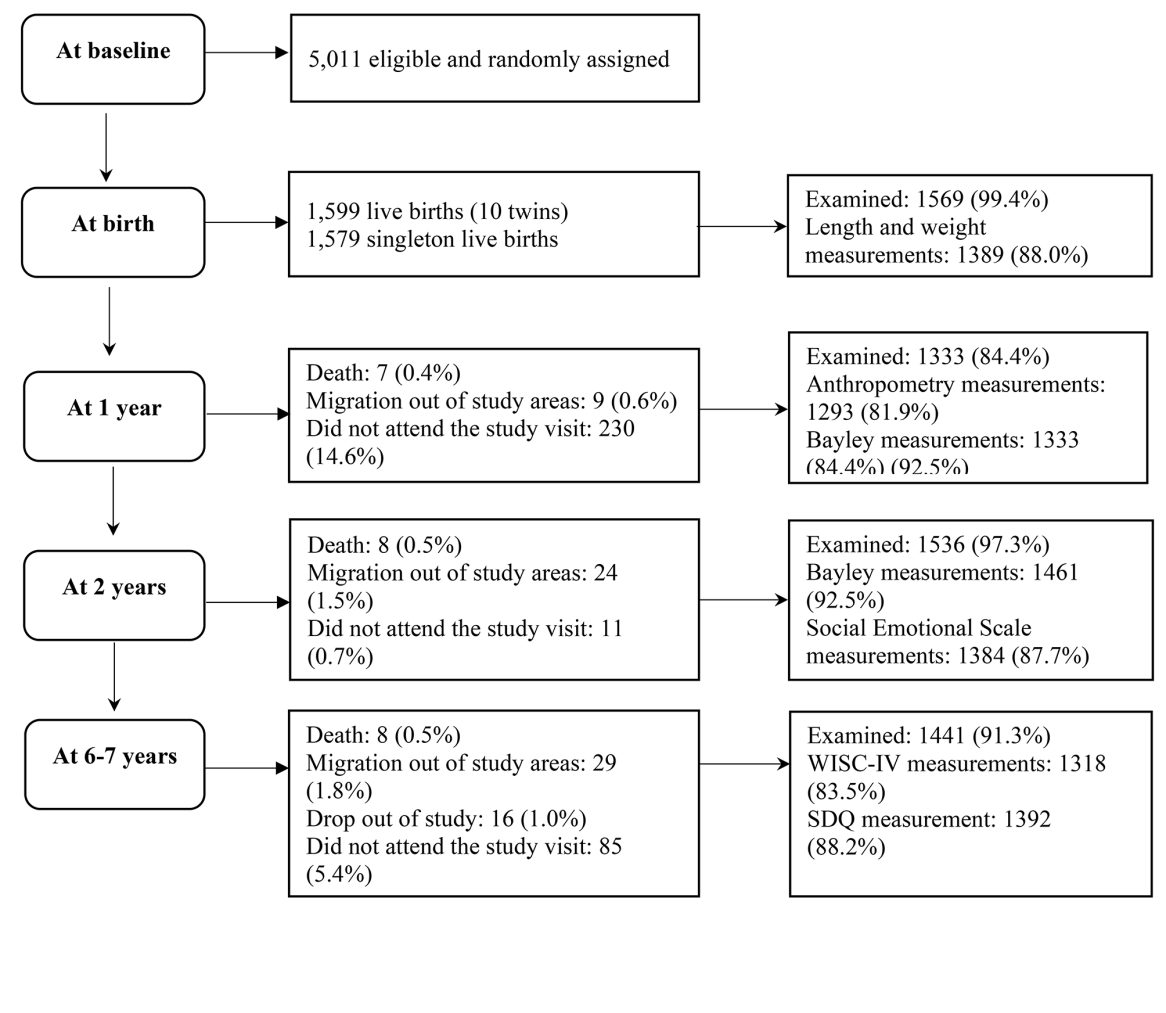
Sample flow WISC-IV: Wechsler Intelligence Scale for Children®—Fourth Edition; SQG: Strengths and Difficulties Questionnaire

This study used data from the Preconception supplementation (PRECONCEPT) study, a double-blind randomized controlled trial to evaluate the effects of preconception micronutrient supplementation on maternal and child health outcomes in Vietnam [23]. In brief, a total of 5011 women of reproductive age from 20 communes in Thai Nguyen province were assigned randomly to receive weekly supplements containing either 2800 µg folic acid (FA), 60 mg iron and 2800 µg FA (IFA), or multiple micronutrients (MM) from baseline until conception, followed by daily IFA (60 mg iron and 400 µg FA) through delivery as a standard of care. Mother-child dyads were followed prospectively from delivery through age 2y and at 6-7y with follow-up rates of more than 84% (Fig. 1). The main reasons for missing data were that participants did not attend the study visits (n = 230, 11 and 85 at 1y, 2y and 6-7y respectively), followed by migration out of the study area (n = 29), dropped out of study (n = 16) and child death (n = 8). The analytic sample for this study included the offspring of women who participated in the PRECONCEPT trial with data on either cognitive or social emotional development at ages 1y (n = 1333), 2y (n = 1461), and 6–7y (n = 1392).

The study was approved by the Ethical Committee of Thai Nguyen National Hospital in Vietnam and Emory University’s Institutional Review Board, Atlanta, Georgia, USA. The trial was registered at ClinicalTrials.Gov as NCT01665378 on 15/08/2012, URL: https://clinicaltrials.gov/ct2/show/NCT01665378. Written informed consent was obtained from all study participants.

### Outcome measures

#### Cognitive development

At 1y and 2y, child cognitive development was measured using the Bayley Scales of Infant Development-III (BSID-III) [24] which includes cognitive, language, and motor subscales. This tool has been translated, validated, and adapted for cultural relevance, with high internal consistency (coefficients ranging from 0.87 to 0.90) and high inter-rater reliability (coefficients ranging from of 0.80–0.94 for different domains) [25].

At 6-7y follow up, child intellectual development was assessed using the Wechsler Intelligence Scale for Children®—Fourth Edition (WISC–IV)  [27] which includes four specific cognitive and language domains (verbal comprehension, perceptual reasoning, working memory and processing speed), and an overall Full-Scale Intelligence Quotient (FSIQ). The tool has been validated and adapted in the Vietnamese context, including the translation, cultural analysis, modifications, and standardization . Particularly, the tool was translated into Vietnamese and back translated by a group of bilingual psychologists and health researchers. The Adaptation Committee, including four members who are child clinical psychologists and understand cultural and linguistic of origin of the WISC-IV (the U.S.) and from Vietnam, provided overall guidance for the adaption process. Based on a number of culturally-related factors such as linguistic, social and political structures, familiarity with objects, problematic items and item ordering, which were identified from the piloted and standardized studies, the Adaptation Committee revised the tools for cultural appropriateness [28].

All measures of cognitive development were directly administered by well-trained researchers in a quiet room at community health centers in the presence of the caregiver. Raw summary scores for each of the subtests were computed as described in the test manuals and then transformed to composite scores (with mean ± SD of 100 ± 15, ranged 40–160). Average scores of the cognitive and language domains obtained from BSID-III were generated as a composite measure of cognitive development at ages 1y and 2y to compare with the FSIQ – a combination of cognition and language domains at 6-7y from the WISC-IV.

#### Social-emotional development

Child socio-emotional development was measured based on caregiver report using the BSID-III tool at 1y and 2y, and the Strengths and Difficulties Questionnaire (SDQ) at 6-7y. Both BSID-III and SDQ tools have been validated and widely used to assesses emotional and behavioral disorders among children in several countries including Vietnam [26] [41]. The reliability, validity, and incremental validity of Vietnamese versions of the SDQ were evaluated in a community sample of children ages 6 to 16 years old from 10 Vietnamese provinces, as well as in a clinical sample of inpatients and outpatients at the same age from three psychiatric facilities in Hanoi [26]. The Vietnamese version of the SDQ-P is available as a free download from www.sdqinfo.com. The social emotional scale at 2y was constructed as a sum of responses for 24 questions and then standardized over ages based on BSID-III guideline [24], whereas at 6-7y, it was calculated by aggregating five items of prosocial behavior subtest.

All the outcome measures were finally transformed to standardized Z-scores to facilitate comparisons of child’s performance over time within and between individuals.

### Wealth index

A household wealth index was constructed for all children/households at 1y, using a principal component analysis which included several variables related to house and land ownership, housing quality, access to services (electricity, gas, water, and sanitation services), and household assets (productive assets, durable goods, animals, and livestock). The first component derived from component scores explained 40% of the variance and was then used to categorize wealth into quintiles where the lowest quintile (Q1) represents the poorest 20% and the highest quintile (Q5) represents the richest 20% of the population [27]. The wealth gap was defined as the gap between the lowest and the highest quintile. Since changes in wealth status may also influence our outcomes, we also constructed the household wealth index based on data that were collected when the children were 6-7y. We used the residual of wealth index in middle childhood as a measure of change in wealth status by regressing the measurement of wealth at 6-7y on this measurement at 1y.

### Potential mitigating factors

*The quality of the learning environment at home* was measured using the Infant/Toddler HOME inventory at 1y and the Middle Childhood HOME Inventory at 6-7y []. Specifically, the HOME assesses the quality and quantity of stimulation and support available to a child in the home environment including parental responsivity, encouragement of maturity, acceptance of the child, organization of the environment, learning materials, parental involvement, variety, and physical environment. Because of the correlation between repeated measures of home environment quality for each child at 1y and 6-7y, we created the residual from a linear regression of the middle childhood measure on the earlier childhood measure.

*Maternal factors* including education, intelligence, and depression were used as other potential mitigating factors. Education was recorded as the highest grade that the mother attended, and maternal intelligence quotient (IQ) was assessed using the Ravens Progressive Matrices [28] at 1y. Maternal depression was measured by the Center for Epidemiologic Studies Depression Scale when the child is at 1 year [29]. We did not include marital status as more than 96% were married. Similarly, as very few reported the use of tobacco (1%) or alcohol (< 5%) these were not included as control variables [30, 31].

For *child nutritional status*, we used child height-for-age-z-scores (HAZ) at 1y which is a cumulative indicator of linear growth that reflects prolonged nutrition during childhood [32]. We also used stunting, that is defined as HAZ below two standard deviations from the WHO growth reference (HAZ<-2) and is a widely accepted as marker of chronic malnutrition [33].

*Child school attendance* was measured by asking mothers for how long their children had attended day care centers and pre-schools when the children was less than 36 months and 36–72 months respectively. The enrollment was defined as a binomial variable of attendance at least one year in each period (Yes/No).

*Other control variables* included ethnicity, types of preconception supplementation, child age, and child sex.

### Statistical analysis

Descriptive analyses (means or percentages and standard deviations) were used to report characteristics of the study population. The wealth gaps in cognitive and social-emotional scores over time were visualized separately for boys and girls. Multivariable linear regression analyses were used to assess the association of wealth quintile with child development. To assess the roles of potential mitigating factors, we estimated the size of the wealth gaps for the lowest and highest quintiles in five sequential steps: In step 0, we included only wealth as predictor variable (unadjusted model). In step 1, we included the quality of home environment score in early childhood (for outcomes at 1y and 2y) and the home environment in both early and middle childhood (for outcomes at 6-7y). In step 2, we included additional maternal factors (education, IQ, and depression). In step 3, we added child HAZ at 1y as an indicator of early child nutrition. Finally, in step 4 we added school attendance that was measured in middle childhood. We adjusted for ethnicity, types of preconception supplementation, child age and child sex at all steps. Bootstrap methods [34] with 1000 replications were used to test whether the set of potential mitigating factors introduced in each new step significantly modified the size of wealth gap compared to the previous step. For each step, we computed the relative change in the wealth gap (by dividing the change in the wealth gap in this step over the size of the gap in the previous step). We used the Bootstrap method to test differences in the size of the gaps for child development over time. Finally, we also conducted stratified analyses by child sex and risk of stunting (HAZ <-1) at 1y. We applied the Bonferroni correction for all P values to account for multiple testing. All statistical analyses were conducted in STATA software version 17.

## Results

Selected maternal and offspring characteristics at 1y, 2y, and 6-7y for the study sample are presented in Table 1. Average maternal age at enrollment was 26 years old and nearly 50% of women were ethnic minorities. Mothers had about 10 years of schooling on average. The home quality environment score was 63.3 (SD 8.3, range 0-100) at 1y and 56.0 (SD 12.6) at 6–7. There were an equal number of boys and girls. Mean HAZ was − 0.87 (SD 1.04) and 13.1% of children were stunted at 1y. Only 11% of children attended preschool at least one year while most of them (97%) enrolled in kindergarten.

**Table 1:** Sample characteristics

Variable	Mean ± SD or %
Maternal characteristics	
Age at baseline, y	25.87 ± 4.33
Ethnic minority	49.4
Schooling, y	9.66 ± 2.92
IQ score	86.60 ± 16.92
Depressive score	1.91 ± 3.06
Types of preconception supplementation, %	
FA	32.80
IFA	32.36
MM	34.84
**Household characteristics**	
Wealth index	0.02 ± 0.95
Home quality environment score at 1y (range 0-100)	63.28 ± 8.32
Home quality environment score at 6-7y (range 0-100)	56.00 ± 12.60
**Child characteristics at 1y measurement**	
Age, *m*	12.89 ± 0.74
Girls, *%*	49.81
HAZ	-0.87 ± 1.04
Stunting, *%*	13.07
**Child characteristics at 2y measurement**	
Age, *m*	24.41 ± 0.90
Girls, *%*	49.54
**Child characteristics at 6-7y measurement**	
Age, *m*	77.45 ± 3.93
Girls, *%*	48.46
Attend preschool at least 1y, %	10.71
Attend kindergarten at least 1y, %	96.77

Mean scores for each domain and overall cognitive and social-emotional development at 1y, 2y, and 6-7y are presented in Table 2. There was no significant difference in overall cognitive score between boys and girls at 1y or 6-7y, but girls had significantly higher cognitive (2.1 points) and social emotional score (0.7 points) compared to boys at 2y. Overall FSIQ score at 6-7y was 88 (SD 12.2, ranged 41–126), and average raw score of prosocial behavior was 6.0 (ranged 0–10).

**Table 2:** Cognitive and social-emotional development, by child sex

	Girls	Boys
At 1 year (n = 1,333)		
*BSID-III, scaled scores*		
Cognitive	112.0 ± 10.5	112.4 ± 10.0
Language	98.4 ± 11.0**	96.8 ± 11.0
Overall	105.2 ± 9.0	104.6 ± 8.7
**At 2 years (n = 1,461)**		
*BSID-III, scaled scores*		
Cognitive	100.4 ± 10.0**	98.8 ± 9.8
Language	103.5 ± 10.3***	101.1 ± 11.2
Overall	102.0 ± 9.1***	99.9 ± 9.5
*Social emotion*	9.3 ± 3.6***	8.6 ± 3.2
**At 6–7 years (n = 1,318)**		
*WISC-IV, scaled scores*		
Verbal comprehension	81.5 ± 12.0	82.0 ± 12.8
Perceptual reasoning	93.0 ± 14.0	93.2 ± 14.9
Working memory	102.7 ± 11.1**	101.0 ± 11.9
Processing speed	90.2 ± 11.9*	88.6 ± 12.6
Overall FSIQ	88.5 ± 11.7	88.0 ± 12.5
*Social emotion*	6.0 ± 2.9	5.8 ± 2.8

*p < 0.05; ** p < 0.01; *** p < 0.001

**Fig. 2:**
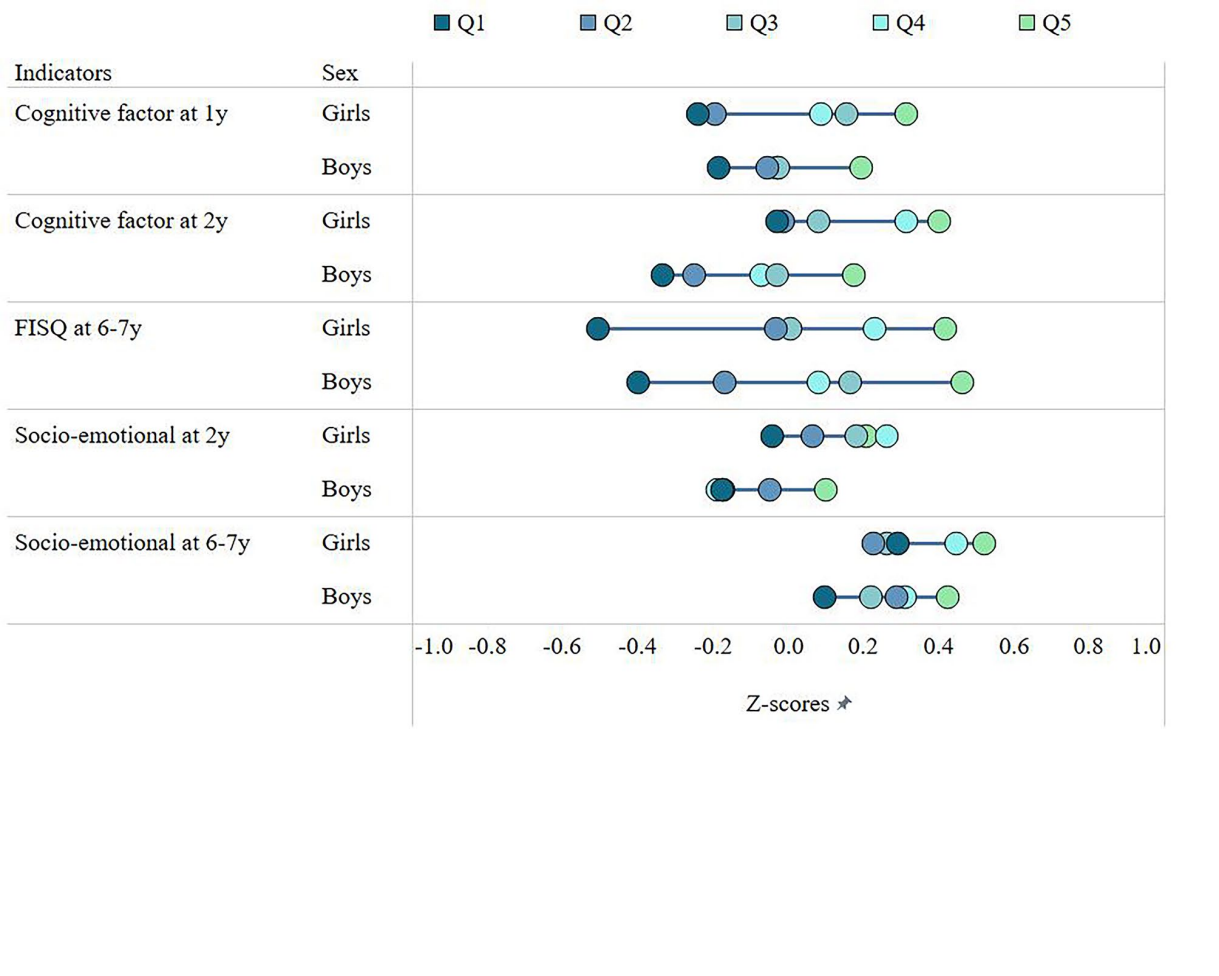
Inequality trends in child development, by wealth quintile FSIQ: Full-Scale Intelligence Quotient; Q: Quintile

The wealth gaps in cognitive and social-emotional development across these periods are shown in Fig. 2. For cognitive development, the size of wealth gaps between the lowest and highest quintiles at 1y and 2y were similar, both being about 0.5 SD. This gap nearly doubled for FISQ at 6-7y (0.9 SD). We also found similar but smaller wealth gaps for social-emotional development at both 2y and 6-7y where being in the highest quintile was associated with significantly higher social-emotional score compared to the lowest quintile (0.25 SD and 0.30 SD respectively), but these gaps remained unchanged over time. Finally, girls had higher scores in some development indicators, although there were no differences in the wealth gaps by sex in all study periods.

**Fig. 3:**
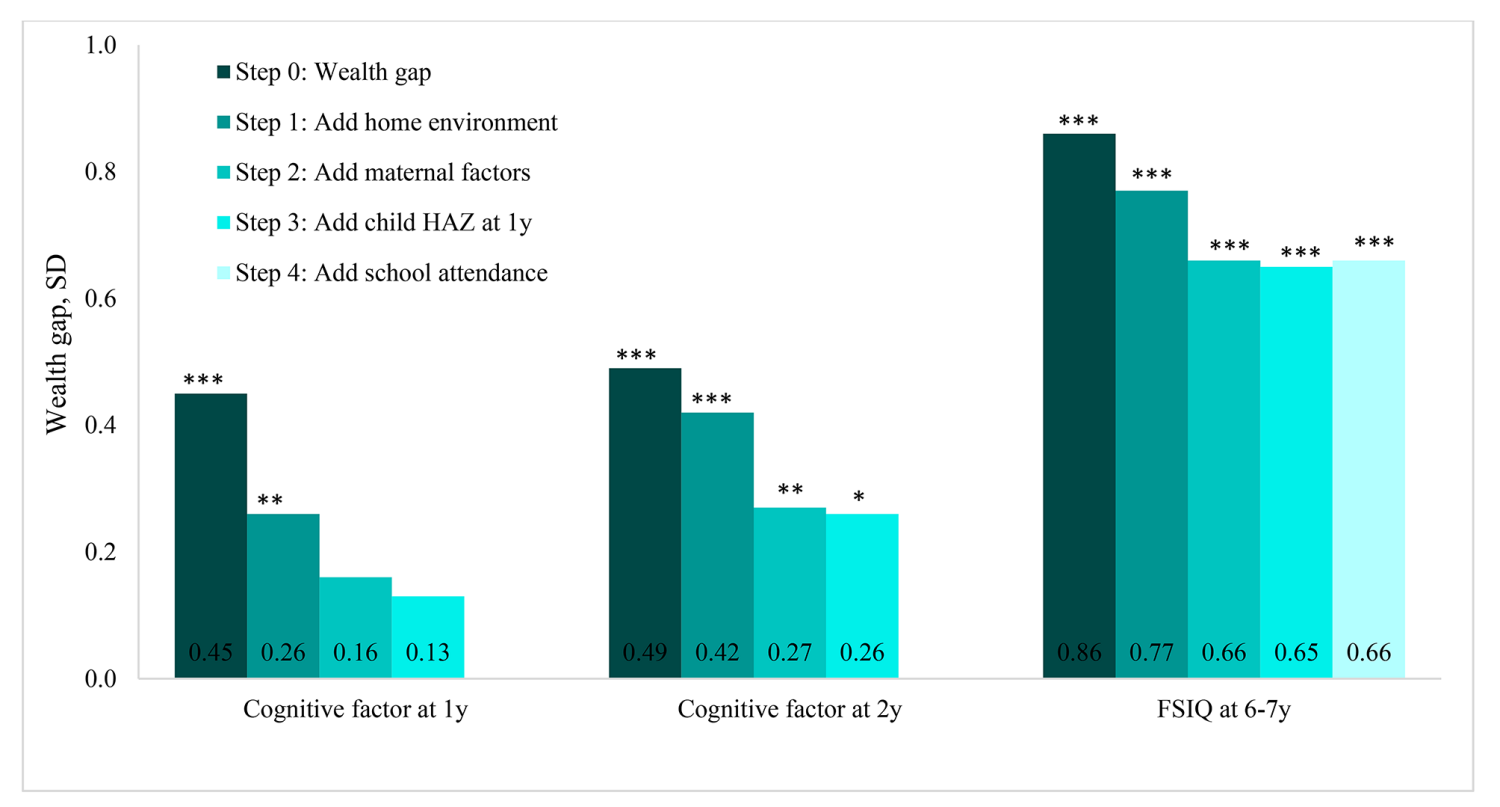
Wealth disparities in cognitive development The comparisons were between the first and the fifth quintiles. Step 1 was adjusted for maternal ethnicity, child age, and child sex. Other steps adjusted for the controlled variables in step 1 and types of preconception supplementation. Cognitive development at 6-7y models also adjusted for wealth residual at 6-7y. Maternal factors including education, IQ, and depression Statistical significance from multivariable linear regression in each step comparing difference in outcomes between the lowest and highest wealth quintiles: * p < 0.05, ** p < 0.01, *** p < 0.001 FSIQ: Full-Scale Intelligence Quotient; HAZ: Height-for-age-z-scores; SD: Standard deviation

Figure 3 presents the significant effects of potential mitigating factors in reducing the wealth gaps in cognitive development at 1y, 2y and 6-7y between the lowest and highest quintiles from multivariable regression analyses. At 1y, the unadjusted wealth gap was 0.45 SD; the gap reduction was largest when entering the quality of home environment (by 42.2%, p < 0.001), followed by maternal factors (by 38.5%, p < 0.01), and child HAZ (by 18.8%, p < 0.01) (Additional file 1). At 2y, maternal factor was the main mitigating factor that significantly reduced the wealth gap by 33% (p < 0.001), while the quality of home environment accounted for the reduction of the wealth gap by 14%. The wealth gap in cognitive at 2 y, however, still persisted despite the important contribution of mitigating factors. At 6–7 y, the reductions in the wealth gap were similar to that at 2y but the contributions of mitigating factors were much smaller; the quality of the home environment reduced the wealth gap by 10.5%, while maternal factors accounted for 14% of the reduction. Child HAZ at 1y or school attendance did not affect the wealth gap for FSIQ. At all three time points, we did not find any effects of the preconception micronutrient supplementation in either increasing or decreasing the wealth gap (results not shown). Stratified analysis did not show differences in the wealth gaps in cognitive development by sex or risk of stunting (HAZ < -1) (results not shown). Ethnicity was not associated with child development and did not contribute to the size of wealth gaps in these outcomes (results not shown).

**Fig. 4:**
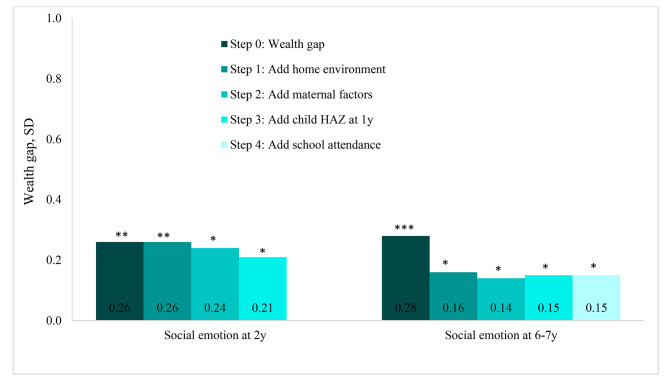
Wealth disparities in social-emotional development The comparisons were between the first and the fifth quintiles. Step 1 was adjusted for maternal ethnicity, child age, and child sex. Other steps adjusted for the controlled variables in step 1 and types of preconception supplementation. Social emotion at 6-7y models adjusted for wealth residual at 6-7y. Maternal factors including education, IQ, and depression Statistical significance from multivariable linear regression in each step comparing difference in outcomes between the lowest and highest wealth quintiles: * p < 0.05, ** p < 0.01, *** p < 0.001 HAZ: height-for-age Z-score; SD: Standard deviation

The changes in wealth gaps in social-emotional development are shown in Fig. 4 and Additional file 2. At age 2y, this wealth gap was 0.26 SD and reduced slightly when we controlled for maternal factors (7.7%), but reduced more substantially whereas including child HAZ (12.5%). At age 6-7y, the inclusion of the quality of home environment significantly reduced the size of wealth gap by 43%. The inclusion of additional factors reduced wealth gap by about 13%, but the remaining gap was still significant. Supplements during preconception did not contribute to the reduction of wealth gap in social emotion at both 2y and 6-7y. There were no differences in wealth gaps when stratified by sex or stunting (results not shown).

## Discussion

We found evidence of significant differences in child development between the richest and poorest population in Vietnam at both early and middle childhood. The wealth disparity in cognitive development was apparent as early as 1 year of age, maintained at 2y and increased remarkably at 6-7y, whereas the gap in social emotional development did not change over time. Our findings also clearly demonstrated that the quality of the home environment, maternal factors, and child HAZ significantly mitigated the disparities in cognitive development at early childhood and contributed to a small reduction of the gap at middle childhood. Most notably, the quality of the home environment played a key role in reducing the wealth gap in about 43% of cognitive development at 1y, and 50% of prosocial behavior score at 6-7y. The absence of any impact of the preconception intervention on the wealth gap, implied that there was neither evidence of a reinforcing interaction with wealth over time nor one that diminished such gaps. Thus, the intervention appeared neutral with respect to wealth.

In line with the existing evidence, inequity in child cognitive development by wealth quintile appears from early childhood and persists through early schooling. Results from a longitudinal study in Bangladesh [21] and cross-sectional survey in India, Indonesia, Peru, and Senegal [17] showed that the wealth gap ranged from 0.2 SD to 0.7 SD and was apparent as early as 7 months through 16–23 months of child age. Wealth disparity was also found over a period of 3-8y in longitudinal surveys of Madagascar [18], Colombia [20], and in the Young Lives Study of 4 low and middle income countries [16] with magnitudes ranging from 0.6 SD to 1.5 SD. These wealth gradients in child development, nevertheless, are country-specific and vary with population demographic, health, and socioeconomic characteristics [35]. While the size of the wealth gap in cognitive development was unchanged as the child aged in some studies [15, 16, 36], it was wider at middle childhood in our study and in other developing countries such as Madagascar, India, Indonesia, Peru and Senegal [17, 18]. This is probably due to the inequalities in child development beginning prenatally and the exposure with risks was multiple and cumulative [2].

Overall, home quality environment and maternal factors were consistently the most important mitigating factors of the wealth gap in cognitive development, while child growth and attendance to school are heterogeneous across countries and ages. Our findings showed that home stimulation reduced 10 − 42% of the observed wealth gap in cognitive development, which was aligned with a range of 18 − 37% documented in literature [17, 20, 21]. Similar to findings of previously in studies conducted in low- and middle-income countries [16, 37] we also found that the magnitude of disparity declined significantly after adding maternal education. Given global trends in expanding female education this is an encouraging observation.

While previous studies included only maternal education, we were able to examine the effects of maternal IQ and mental health which were strongly associated with child development in our analysis. The mitigating effects of early child physical growth (measuring at different time points) on disparity in child cognitive development also differ over time across countries studied in the literature. For example, HAZ was a significant mediator in reduction of 10–20% wealth gap in Indonesia, Senegal, and India at early childhood [17], but it did not influence the wealth disparity in other countries at middle childhood [16, 20, 21]. Combining effect of home quality environment, maternal factors, and early child physical growth reduced 70% wealth gradient at 1y and 47% at 2y, consistent findings with the reduction of major wealth gradient in Peru, India, Vietnam, and Bangladesh [16, 21]. The impact of attendance in preschool and kindergarten on child development is variable; positive effects have been reported in some studies but no impacts have been observed in others [37], depending on types of public or private school, quality of program and curricula, trained teaching staff and classroom site. Our study did not find a mitigating effects of school attendance, partly due to less heterogeneity in the data. Only a small proportion (~ 11%) attended public preschool while virtually all of the children (97%) attended public kindergarten at the appropriate age. A previous study also found high overall attendance in Vietnam and a comparatively small gap between the poorest and wealthiest quintiles^16^,^1^ . The small reduction of the wealth gap in cognitive measurement (22%) at middle childhood after controlling for all invested factors implied that additional factors such as quality of early education and peer influence should be investigated.

The relatively small gaps in social emotional developments were similar in magnitude (0.2–0.3 SD) to the results reported in the Colombia [38] and Madagascar samples [18]. In line with the result in the Colombia study, the wealth gap in social emotional developments did not evolve appreciably over time [38]. Contributions of home environment, maternal factors, and nutritional status in reducing the disparity varied across study periods. At 2y, child HAZ contributed a small amount to narrowing the wealth gap, while home quality environment reduced the largest proportion of the changes in the gap by 6-7y. The results are suggestive of child growth and support from home operating differentially and independently on social emotional development.

This study has some important limitations. Since the study was conducted in rural area, we are not able to compare difference between rural and urban setting as well as the contribution of residence area in wealth disparity. We did not however observe any differences associated with ethnicity in this rural population. Social emotional development was also measured by caregiver reports, which might be influenced by culture and may be affected by recall bias. Although we also did not measure maternal trauma which could influence child cognitive and social emotional development, we had measures of depression that were extremely low [39]. Although we did not examine the role of specific nutrient deficiencies and prior research has shown positive long-term impacts of preconception micronutrient supplementation, this intervention did not appear to have an impact on wealth disparities.

In conclusion, our study finds significant wealth disparities in child development in Vietnam. The quality of the home environment, maternal factors, and child HAZ were important mitigating factors of the disparities in cognitive development and represent potential areas of intervention for reducing wealth disparities in this population. Our findings have important public health and policy implications. Specifically, there is an urgent need to develop and implement multidisciplinary poverty alleviation strategies that are targeted to benefit families with young children. Special attention on improving the quality of home environment, investments in education, and improving access to nutritious food and quality health care especially during the critical windows namely the first 1000 days is needed to ensure that all children meet their full developmental potential.

## Electronic supplementary material

Below is the link to the electronic supplementary material.

Supplementary Material 1

Supplementary Material 2

## Data Availability

All data analyzed during this study are included in this published article [additional file 3].

## Abbreviations

BSID-III: Bayley Scales of Infant Development-III
FA: Folic acid
FSIQ: Full-Scale Intelligence Quotient
HAZ: Height-for-age-z-scores
IFA: Iron and folic acid
IQ: Intelligence quotient
MM: Multiple micronutrients
PRECONCEPT: Preconception supplementation
Q: Quintile
SD: Standard deviation
SDQ: Strengths and Difficulties Questionnaire
WISC–IV: Wechsler Intelligence Scale for Children®—IV
