# Supplementary material for: Home environment and nutritional status mitigate the wealth gap in child development: a longitudinal study in Vietnam
Source: BMC Public Health. 2023 Feb 8;23:286. doi: 10.1186/s12889-023-15156-2 (PMC9906900; doi:10.1186/s12889-023-15156-2)
Supplement: Supplementary file 1 — Supplementary Material 1 [file 12889_2023_15156_MOESM1_ESM.docx]

**Additional file 1: Changes in wealth disparities in cognitive development due to potential mitigating factors**

|  | **Cognitive factor at 1y** | | **Cognitive factor at 2y** | | **FSIQ at 6-7y** | |
| --- | --- | --- | --- | --- | --- | --- |
|  | **β [95%CI]** | **Relative change (%)** | **β [95%CI]** | **Relative change (%)** | **β [95%CI]** | **Relative change (%)** |
| Step 0: Wealth index | 0.45*** [0.27,0.63] |  | 0.49*** [0.30,0.66] |  | 0.86*** [0.68,1.05] |  |
| Step 1: Add home environment | 0.26** [0.08,0.45] | 42.2*** | 0.42*** [0.23,0.61] | 14.3 | 0.77*** [0.57,0.98] | 10.5* |
| Step 2: Add maternal factors | 0.16 [-0.03,0.36] | 38.5** | 0.27** [0.07,0.48] | 33.3*** | 0.66*** [0.45,0.87] | 14.3*** |
| Step 3: Add child HAZ at 1y | 0.13 [-0.07,0.33] | 18.8** | 0.26* [0.06,0.46] | 7.1 | 0.65*** [0.44,0.86] | 1.5 |
| Step 4: Add school attendance |  |  |  |  | 0.66*** [0.44,0.87] | -1.5 |

All steps adjusted for child age, child sex, ethnicity and types of preconception supplementation. Cognitive development at 6-7y models also adjusted for wealth residual at 6-7y. Maternal factors including education, IQ, and depression.

Statistical significance from multivariable linear regression in each step: * p< 0.05, ** p < 0.01, *** p< 0.001

FSIQ: Full-Scale Intelligence Quotient; HAZ: height-for-age Z-score;
