# Supplementary material for: Home environment and nutritional status mitigate the wealth gap in child development: a longitudinal study in Vietnam
Source: BMC Public Health. 2023 Feb 8;23:286. doi: 10.1186/s12889-023-15156-2 (PMC9906900; doi:10.1186/s12889-023-15156-2)
Supplement: Supplementary file 2 — Supplementary Material 2 [file 12889_2023_15156_MOESM2_ESM.docx]

**Additional file 2: Changes in wealth disparities in social-emotional development due to potential mediators**

|  | **Social emotion at 2y** | | **Social emotion at 6-7y** | |
| --- | --- | --- | --- | --- |
|  | **β [95%CI]** | **Relative change (%)** | **β [95%CI]** | **Relative change (%)** |
| Step 0: Wealth index | 0.26** [0.08,0.43] |  | 0.28*** [0.16,0.40] |  |
| Step 1: Add home environment | 0.26** [0.07,0.44] | 0 | 0.16* [0.03,0.29] | 42.9*** |
| Step 2: Add maternal factors | 0.24* [0.04,0.43] | 7.7 | 0.14* [0.01,0.28] | 12.5 |
| Step 3: Add child HAZ at 1y | 0.21* [0.02,0.41] | 12.5*** | 0.15* [0.01,0.28] | -7.1 |
| Step 4: Add school attendance |  |  | 0.15* [0.01,0.28] | 0 |

All steps adjusted for child age, child sex, ethnicity, and types of preconception supplementation. Social emotion at 6-7y models adjusted for wealth residual at 6-7y. Maternal factors including education, IQ, and depression.

Statistical significance from multivariable linear regression in each step: * p< 0.05, ** p < 0.01, *** p< 0.001

HAZ: height-for-age Z-score;
